# ^99m^Tc-TRODAT-1 SPECT Imaging in Early and Late Onset Parkinson’s Disease

**DOI:** 10.22038/aojnmb.2017.8844

**Published:** 2017

**Authors:** Payam Sasannezhad, Ali Ghabeli Juibary, Kayvan Sadri, Ramin Sadeghi, Mahsa Sabour, Vahid Reza Dabbagh Kakhki, Hesam Alizadeh

**Affiliations:** 1 Department of Neurology, Mashhad University of Medical Sciences, Mashhad, Iran; 2 Nuclear Medicine Research Center, Mashhad University of Medical Sciences, Mashhad, Iran

**Keywords:** Dopamine transporter, Early onset, Late onset, Parkinson’s disease, ^99m^Tc-TRODAT-1

## Abstract

**Objective(s):**

^99m^Tc-TRODAT-1, which binds to the dopamine transporter, could be used to image the dopaminergic system in diagnosis of Parkinson’s disease (PD). PD can be classified into two groups: late onset Parkinson’s disease (LOPD) and early onset Parkinson’s disease (EOPD). In this study we tried to determine the TRODAT SPECT findings in EOPD as compared to LOPD.

**Methods:**

Fifteen patients were studied. The diagnosis of PD was defined by clinical criteria based on UK Parkinson’s Disease Society Brain Bank criteria. Six patients whose age at onset of PD were younger than 50 were defined as patients with EOPD and 9 patients with older than 50 years were defined as patients with LOPD. All patients underwent ^99m^Tc-TRODAT Brain SPECT.

**Results:**

There was a significant decrease of striatal ^99m^Tc-TRODAT-1 (TRODAT) binding in PD patients in both EOPD and LOPD. No significant difference was noticed between EOPD and LOPD in disease stage and symptoms. In visual analysis, 20 (66.67%) caudate nucleuses had decreased tracer uptake while all 30 (100%) putamens had decreased or absent tracer uptake. No significant difference between EOPD and LOPD was noticed in visual analysis. Striatum, Caudate and Putamen uptake ratio to background were calculated. No significant difference was noticed between EOPD and LOPD in these ratios. However there was significant difference in visual analysis (tracer uptake) as well as in uptake ratio between putamen and caudate nucleuses in both groups (P=0.001). On the other word, we found more diminished uptake in putamen as compared the caudate. Frequency and severity of putamen involvement were much more than caudate.

**Conclusion:**

^99m^Tc-TRODAT-1 SPECT imaging showed lower presynaptical dopami-nergical terminals density in both EOPD and LOPD. There was no difference between EOPD and LOPD in TRODAT uptake. Putamen showed more involvement and more diminished TRODAT uptake.

## Introduction

Parkinson’s disease (PD), a common neuro-logical disorder, is primarily associated with a progressive degeneration of dopaminergic neurons in the nigrostriatal pathway (1, 2). Accurate diagnosis of Parkinson’s disease is vital. Now a day, the diagnosis of Parkinson’s disease is still based on clinical criteria which can be incorrect especially in early stages (1-3). PD was seen about in 2% of persons over 60 years (1). PD could also be seen earlier in life as early onset PD (EOPD) which defined in different ages from below 40 years of age up to 58 years (1, 4, 5). In vivo imaging of the dopaminergic system can improve the diagnosis of PD (3). SPECT and PET imaging using radioligands with high affinity to the dopaminergic system could be used in diagnosis of PD, determining degenerative or non-degenerative forms of parkinsonism and estimating dopamine cell loss (6).

One of the most important dopaminergic binding sites is the dopamine transporter (DAT), which is located in the presynaptic membrane on the terminal of the dopaminergic projection and it’s responsible for the re-uptake of dopamine (2, 7, 8). DAT is a marker of dopamine terminal innervation. Thus, in vivo DAT imaging may provide a measure of dopamine terminal innervation of the striatum (2). Technetium-99m labeled tropane derivative, ^99m^Tc-TRODAT-1, which binds to the dopamine transporter, could be used to image the dopaminergic system (3, 9, 10). It is reported that there is a close relationship between DAT concentrations and striatal dopamine levels (2, 11, 12). ^99m^Tc-TRODAT-1 (TRODAT) SPECT could provide an ideal tool for evaluation of PD (1). Some previous studies demonstrated that TRODAT uptake is diminished in the striatum of patients with PD and TRODAT SPECT imaging can discriminate between PD and healthy volunteers (3). PD can be classified into two groups: late-onset Parkinson’s disease (LOPD) that characteristically begins in older patients (such as after 50 years of age) and early onset Parkinson’s disease (EOPD) that occurs in early life (between 21 and 50 years of age). Five percent of all PD are EOPD which has slower disease progression (13).

In this study we tried to determine the TRODAT SPECT findings in EOPD as compared to LOPD.

## Methods

### Patients

Fifteen patients (mean age: 54.6±13.29; 36-82 years) were studied. The diagnosis of PD was defined by clinical research criteria based on UK Parkinson’s Disease Society Brain Bank criteria (14, 15). Based on reference criteria, Parkinson-like disease were excluded from the study. The severity and disability level of Parkinson’s disease was assessed using the Hoehn and Yahr scale (H& Y) (16).

Patients with systemic disease, depression, neuropsychiatric disorders, dementia, secondary or symptomatic Parkinsonism, such as PD induced by drugs or environmental toxins, or Parkinson-plus syndromes, such as progressive supranuclear palsy (PSP), or multiple system atrophy (MSA), were excluded from this study.

Six patients whose age at onset of PD were younger than 50, were defined as patients with EOPD and 9 patients with older than 50 years were defined as patients with LOPD.

The study was approved by local ethical committee. Informed consent was obtained from all patients.

### ^99m^Tc-TRODAT Brain SPECT

**Figure 1:**
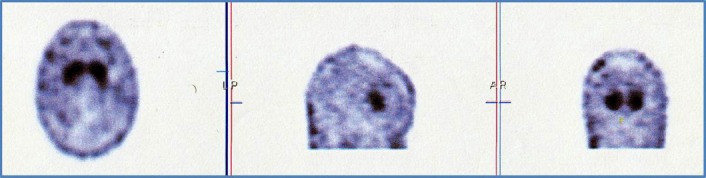
Normal SPECT images of 99mTc-TRODAT-1 imaging

Four hours after intravenous injection of a single bolus injection of 740 MBq ^99m^Tc-TRODAT-1, SPECT imaging was performed. The brain SPECT images were acquired using a dual-head gamma camera equipped with parallel hole, high resolution-low energy collimators (E.Cam, Siemens). Data were acquired in a 128 × 128 matrix with a 1.4 zoom through 360° (180° for each head) rotation on the step and shoot mode (stop on time per projection=30 s). Images were reconstructed using backprojection with a Metz filter. Attenuation correction was accomplished using Chang’s first order correction method. The SPECT images were analyzed visually and semi-quantitatively (Figure 1).

Based on amount of uptake visually, striatum uptake were divided in to three groups: Normal (significantly higher than background uptake), Absent (equal to background) and Decreased TRODAT uptake (slightly more than background).

One experience nuclear medicine specialist draw regions of interest around the whole striatum, putamen, caudate and occipital cortex (OC) of each hemisphere. He was blind about the diagnosis: EOPD or LOPD. The regions of interest were drawn based on single slice SPECT images with highest uptake in the region of the striatum. The mean uptake ratio for each region was then calculated by dividing the mean activity per pixel in a given ROI by the mean activity per pixel for the reference region using the equation: (mean activity of target ROI – mean activity reference)/mean activity reference. On the other word, the uptake ratio was calculated by subtracting the mean counts per pixel in the OC from the mean counts per pixel in the whole striatum, putamen, or caudate nucleus and dividing the result by the mean counts per pixel in the OC. OC has a low density of DAT, so we used the OC as a reference region (background) (17).

### Statistical analysis

All analyses were done using SPSS 20 software. Data are expressed as mean± SD. Descriptive and frequency statistics, Chi-square analysis testing, Students t test, and Pearson correlation were used to assess associations between the various patients’ characteristics, visual and semi-quantitative variables. Because the data contain small group of patients and the cross tab cells have expected frequencies of less than 5, Exact and Fisher’s Exact test were used. A P value of less than 0.05 was considered statistically significant.

## Results

Patient characteristics were seen in table 1. The mean time between starting the symptoms and diagnosis of PD was 2.1±1.51 years. Mean Hoehn and Yahr stage was 2.13±0.83. Thirty caudate and 30 putamen nucleuses from 15 patients were evaluated. From 6 patients with EOPD, 4 patients were in stage 2 and 2 patients were in stage 3. From 9 patients with LOPD; 3, 4, 1 and 1 patients were stage 1, 2,3, and 4 respectively. There was no significant difference between EOPD and LOPD in disease stage and symptoms. In visual analysis, 20 (66.67%) caudate nucleuses had decreased tracer uptake while all 30 (100%) putamens had decreased or absent tracer uptake. No significant difference was noticed between EOPD and LOPD in visual analysis (Table 2). Table 3 showed Striatum, Caudate and Putamen uptake ratio. No significant difference was noticed between EOPD and LOPD in these ratios (P>0.05). No significant difference was noticed between EOPD and LOPD in visual analysis and semi-quantitative analysis based on disease stage. However there was significant difference in visual analysis (tracer uptake) as well as in uptake ratio between putamen and caudate nucleuses (Table 2 and Table 3; P=0.001). There was no any adverse effect in either patient during and after imaging.

**Table 1:** Patients’ characteristics: age, sex, family history of Parkinson’s disease (PD) and Hoehn & Yahhr staging in patients with early onset and late onset PD

Early or Late Onset PD	Age	Sex		Positive family history	Hoehn & Yahr
		male	female		
Early Onset PD (6)	42.67±6.62	3	3	1	2.33±0.52
Late Onset PD (9)	62.55±10.19	4	5	3	2.00±1.00
P value	0.001	0.6		0.6	0.4
Total (15)	54.60±13.29	7	8	4	2.13±0.83

**Table 2:** Visual uptake of the Tc99m-TRODAT-1 in putamen and caudate of patients with early onset or late onset of Parkinson’s disease (PD)

Early or Late Onset PD	Tracer Uptake	Left Caudate	Right Caudate	Left Putamen	Right Putamen
Early Onset PD (6)	Normal	2	2	0	0
	Decreased	3	2	3	3
	Absent	1	2	3	3

Late Onset PD (9)	Normal	2	4	0	0
	Decreased	5	4	6	7
	Absent	2	1	3	2

Total (15)	Normal	4	6	0	0
	Decreased	8	6	9	10
	Absent	3	3	6	5

**Table 3:** Semi-quantitative results of uptake ratio ((mean activity of target ROI – mean activity reference)/mean activity reference) in striatum, caudate and putamen of patients with early onset or late onset of Parkinson’s disease (PD)

Early or Late Onset PD	Left Striatum	Right Striatum	Left Caudate	Right Caudate	Left Putamen	Right Putamen
Early Onset PD (6)	0.35±0.22	0.49±0.25	0.62±0.55	0.47±0.39	0.36±0.25	0.25±0.27
Late Onset PD (9)	0.27±0.21	0.45±0.26	0.38±0.29	0.45±0.36	0.29±0.19	0.40±0.36
Total (15)	0.30±0.21	0.47±.25	0.48±0.41	0.46±0.36	0.32±0.21	0.34±0.31

## Discussion

In this study, we have demonstrated that there was a significant decrease of striatal ^99m^Tc-TRODAT-1 (TRODAT) binding in PD patients in both EOPD and LOPD. We didn’t find significant difference in striatal TRODAT uptake between these two groups. On the other hand, we found more diminished uptake in putamen as compared the caudate. Frequency and severity of putamen involvement were much higher than caudate.

Weng et al (17) as well as Mozlev et al (18) demonstrated that there was a significant decrease of striatal TRODAT uptake in PD compared with healthy controls (age-matched). They reported a high sensitivity and specificity of TRODAT uptake values in discriminating PD from healthy subjects (17, 18). Different studies showed that TRODAT SPECT imaging can accurately distinguish patients with early PD and they suggest that TRODAT can be a useful imaging procedure to improve the diagnosis of patients with early symptoms and signs of PD (3, 19, 20). It is reported that there is a close relationship between level of TRODAT uptake and PD severity (1).

In contrast to our findings, Shih MC et al (13) showed EOPD had 34% lower TRODAT uptake than LOPD patients. They suggested patients with EOPD have more dopamine neuronal loss than patients with LOPD. On the other hand, results of some studies are compatible with our findings. Nagasawa et al. (21) found similar levels of striatal ^18^F-dopa uptake between EOPD and LOPD. De la Fuente-Fernandez et al. (22) did not find DAT density difference between EOPD and LOPD groups using ^11^C-Methylphenidate. In a study by ^11^C-FECIT-PET, Antonini et al. (23) concluded comparable diminished of striatal DAT binding in EOPD and LOPD.

In our study we evaluated patients with long lasting disease while the dopaminergical loss of EOPD and LOPD patients in the final stages of the disease may be similar.

Nigrostriatal dopaminergic neurons show greater loss in the putamen than projections to the caudate nucleus, at least in early phases of the PD (1). In our patients, we found more severe and more frequently decrease in TRODAT uptake in putamen as compared to the caudate. Different studies reported that dopamine neurons in the ventral tier of the substantia nigra were most severely affected in PD patients (17). The most loss of DAT binding in the posterior putamen has been well documented (17). Chou et al. (3) also reported an anterior-to-posterior gradient within the striatum for degree of TRODAT uptake. In their study, the posterior putamen had the greatest decrease in specific DAT binding, so it had the greatest ability to distinguish PD patients, while the caudate had the smallest reduction in DAT binding. Different studies suggested that the contralateral putamen is the region in which it can most accurately discriminate between PD patients and healthy controls (3, 24-26). In addition, recent studies showed Striatal ^99m^Tc-TRODAT-1 imaging can be used as a marker for differentiating PD patients from healthy individuals or essential tremor (27, 28). Post-mortem findings also showed a greater depletion of dopamine neurons in the putamen than in the caudate nucleus in the early stages of PD (26, 28). Based on these data, it is evident that TRODAT SPECT imaging is an effective tool for diagnosing and staging of PD. It has be mentioned the striatum and putamen ratios were easier to apply in clinical use than the ratios of the posterior putamen (17).

Study limitations: This study has some limitations, especially due to the small sample size. Age of symptom onset for EOPD definition is controversial (between 40 to 58 years old). It is necessary to perform a large multi-center study with large sample size, perfect matching between EOPD and LOPD, establishing the normal range of the binding ratios in different age groups and determine a high accurate cut-off value of ^99m^Tc-TRODAT uptake ratio for diagnosis of PD.

## Conclusion

^99m^Tc-TRODAT-1 SPECT ima-ging was able to show lower presynaptical dopaminergical terminals density in both EOPD and LOPD. We didn’t find in TRODAT uptake between two groups. On the other hand posterior portion of the striatum (putamen) showed more involvement and diminished TRODAT uptake.
